# Case report: A novel heterozygous frameshift mutation of *ACAN* in a Chinese family with short stature and advanced bone age

**DOI:** 10.3389/fgene.2023.1101695

**Published:** 2023-03-21

**Authors:** Hao Huang, Jieyuan Jin, Rong Xiang, Xia Wang

**Affiliations:** ^1^ Department of Nephrology, Xiangya Hospital, Central South University, Changsha, China; ^2^ Hunan Key Laboratory of Organ Fibrosis, Central South University, Changsha, China; ^3^ National Clinical Research Center for Geriatric Disorders, Xiangya Hospital, Central South University, Changsha, China; ^4^ Department of Pediatrics, Xiangya Hospital, Central South University, Changsha, China; ^5^ Department of Orthopedics, Xiangya Hospital, Central South University, Changsha, China

**Keywords:** short stature, novel mutation, acan, whole-exome sequencing, frameshift, case report

## Abstract

Short stature (OMIM: 165800) is a common pediatric disorder. Any abnormality in the cartilage formation of the growth plate can cause short stature. Aggrecan, encoded by *ACAN*, is an important component of the extracellular matrix. Mutations in *ACAN* have been reported to cause short stature. In the present study, we enrolled a Chinese family with short stature and advanced bone age across three generations. Whole-exome sequencing (WES) was performed on the proband to detect the candidate genes causing short stature in family. A novel heterozygous frameshift mutation (NM_013227.3:c.7230delT; NP_001356197.1: p. Phe2410Leufs*9) of the *ACAN* gene was confirmed to be a genetic lesion in this family. This variant, which was located in a functional site globular 3 (G3) domain of ACAN and predicted to be deleterious by informatics programs, was co-segregated with the affected family members by performing Sanger sequencing. Literatures review of growth hormone (GH) treatment outcome of all previously reported *ACAN* patients suggesting that the G3 domain of ACAN may be critical in the development of short stature and growth hormone treatment. These findings not only contribute to the genetic diagnosis and counseling of the family, but will also expand the mutation spectrum of *ACAN*.

## 1 Introduction

Short stature (OMIM: 165800) is a type of condition that is associated with a child whose height is 2 standard deviations (SD) or more below the mean for children of the same chronological age and sex (Baron et al., 2015; Finken et al., 2018; Grunauer and Jorge, 2018). The growth plate is the target organ of linear growth and height gain (Baron et al., 2015); it is composed of chondrocytes embedded in a collagen matrix that show high metabolic activity. Any abnormality in the cartilage formation of the growth plate can cause linear growth disturbance, which in turn, may lead to short stature.

Existing research suggests that defects in the genes encoding components of either paracrine regulatory systems or the extracellular matrix (ECM), and those encoding intracellular or endocrine proteins result in short stature (Jee et al., 2017). The gene encoding aggrecan (*ACAN*, OMIM: 155760) is located on chromosome 15q26, *ACAN* encodes the production of the proteoglycan aggrecan, which is an important component of the ECM (Stavber et al., 2020). Aggrecan is highly expressed in proliferating chondrocytes of the growth plate. The structural or functional disturbances in aggrecan production could significantly affect cartilage, resulting in changes in linear growth. Statistically, in 1%–5% of idiopathic short stature patients, the short stature can be explained by *ACAN* mutations (Jee et al., 2018).

Herein, we enrolled a Han Chinese family with short stature and advanced bone age (BA). Using whole-exome sequencing (WES) and Sanger sequencing, a novel heterozygous frameshift mutation (NM_013227.3:c.7230delT; NP_001356197.1: p. Phe2410Leufs*9) of the *ACAN* gene was confirmed to be a genetic lesion in this family.

## 2 Case description

**FIGURE 1:**
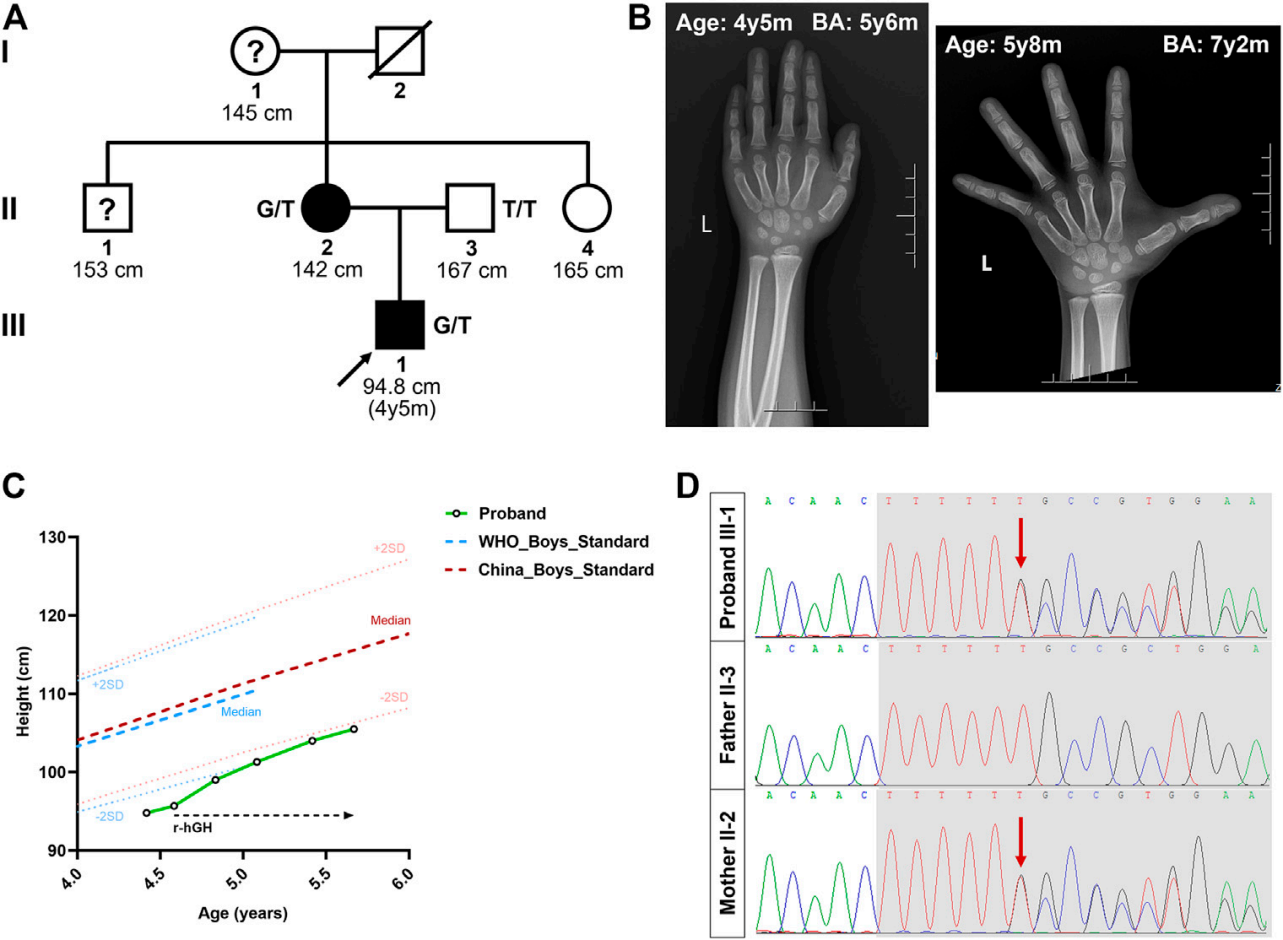
Pedigree of the Chinese family with short stature. **(A)** The black circles/squares represent the affected patients diagnosed with short stature diagnosis; the white circles/squares represent the unaffected subjects. The arrow indicates the proband. The question mark indicates subject with unknown genotype but known phenotype. **(B)** X-ray imaging of the proband’s left hand at the age of 4 years 5 months and 5 years 8 months. **(C)** The height growth curve of the proband during the one-year follow-up. **(D)** The sequencing results of the ACAN mutations. Sequence chromatograms indicate the heterozygosity for a ACAN frameshift mutation (NM_013227.3:c.7230delT; NP_001356197.1:p.Phe2410Leufs*9) in the proband and his mother. The red arrow indicates the mutation. SDS, standard deviation scores; r-hGH, Recombinant human growth hormone; y, year; m, month; BA, bone age.

The proband, a 4-year-old boy, came to the Xiangya Hospital Central South University with his parents due to growth retardation (Figure 1A). Physical examination of the proband showed a height of 94.8 cm (−2.02 SDS), and his weight was 14.6 kg (−1.29 SDS). According to his parents’ recollection, the height of the proband was 70 cm at 12 months of age (−2.40 SDS) and 85 cm at 30 months old (−2.03 SDS). Magnetic resonance imaging revealed a normal size and shape of the pituitary gland. The BA was found to be 5 years and 6 months, as revealed by the X-ray imaging of the left hand (Figure 1B).

We then investigated the proband’s family history. His father (II-3) was 167 cm tall (≈−1.31 SDS), and his mother (II-2) was 142 cm tall (≈−3.23 SDS) (the target height of the proband was 161 ± 5 cm). His mother’s age at menarche was approximately 12 years. Meanwhile, the heights of his grandmother (I-1), maternal uncle (II-1), and maternal aunt (II-4) were 145 cm (≈−2.78 SDS), 153 cm (≈−3.23 SDS), and 165 cm (≈0.28 SDS), respectively (Figure 1A). No other malformations, including intervertebral disc disease, osteochodritis dissecans or back pain, were observed in the affected members of the family.

At the request of his parents, recombinant human growth hormone (GH) treatment was initiated after the proband’s second visit, at a dose of 50 μg/kg/day. During this time, the height of the proband increased to 99.0 cm at 4 years 10 months (−2.17 SDS), 101.3 cm at 5 years 1 month (−1.95 SDS), 104.0 cm at 5 years and 5 months (−1.78 SDS), and 105.5 cm at 5 years and 8 months (−1.76 SDS) (Figure 1C). The growth velocity was also changed from 5.42 cm/year to 6.00 cm/year, after 13 months of GH treatment. Meanwhile, his weight increased to 18.4 kg (−0.52 SDS) and the BA was 8 years during his latest visit, at the age of 5 years 8 months (Figure 1B).

To determine the genetic cause of the short stature in these patients, we extracted the genomic DNA of the proband (III-1) and his mother (II-2). Next, WES was performed to detect the candidate genes associated with short stature and advanced BA. The filtering strategies conformed to those described in our previous study (Huang et al., 2022). Briefly, (a) Variants within intronic, intergenic, and untranslated regions (UTRs), as well as synonymous single nucleotide variants (SNVs), were excluded from later analysis; (b) Common variants were excluded with minor allele frequency ≥0.01 by the 1000 Genomes database, Genome Aggregation Database (gnomAD), version 2.1.1 and an in-house exome database of Berry Genomics; (c) Variants classified as Pathogenic/Likely Pathogenic variants, as well as variant of unknown significance (VUS) by American College of Medical Genetics (ACMG) classification (Richards et al., 2015), could be included; (d) Potential causative variants were then screened by a list of growth-associated genes (Supplementary Table S1) (Hauer et al., 2018); (e) Variants that were not existed in the affected family member (II-2) were excluded; (f) Bioinformatics analysis, including Polyphen-2, MutationTaster, SIFT, and CADD, were also carried out to predict the possible impacts of variants; (g) Accordingly, co-segregation analysis was conducted in the family (Supplementary Figure S1).

After data filtering of (a), (b), (c), (d), and (e), only a set of 7 variants in seven genes in the proband were identified (Supplementary Figure S1, Supplementary Tables S2, S3). Moreover, during further filtering of bioinformatic prediction and co-segregation analysis, a novel heterozygous frameshift mutation (NM_013227.3:c.7230delT; NP_001356197.1: p. Phe2410Leufs*9) in exon 16 of *ACAN* was identified in the proband (Figure 1D). No other significant variant related to short stature or advanced BA was identified. This variant of c.7230delT/p.Phe2410Leufs*9 in the *ACAN* gene was also predicted to be deleterious by multiple bioinformatics software (MutationTaster and CADD).

Sanger sequencing revealed that the c.7230delT/p.Phe2410Leufs*9 variant of *ACAN* co-segregated with the affected family members (I-1, II-1 and II-2). In addition, this novel *ACAN* mutation was not found in the 200 local control subjects from the control cohorts. Based on the ACMG guidelines, the variant of *ACAN* (c.7230delT; p. Phe2410Leufs*9) can be classified as likely pathogenic (PVS1_Moderate + PM1+PM2+PP3).

## 3 Discussion

Short stature is a common clinical manifestation in children. Genetic inheritance is the main determinant of stature (Grunauer and Jorge, 2018). The mutations in *ACAN* gene are a major cause of idiopathic short stature. Aggrecan is a chondroitin sulfate proteoglycan, that is also an important component of the ECM. Aggrecan is encoded by *ACAN*. Animal experiments shown that dysfunction of the chick and mouse aggrecan can lead to severe chondrodystrophies with perinatal lethality (Li et al., 1993). Moreover, the expression levels of important regulatory genes, including *Col10a1*, *Sox9*, and *Ihh*, were sharply reduced in *ACAN* knockout mice (Lauing et al., 2014). In humans, mutations in the *ACAN* gene are known to cause chondrodysplasia and inherited short stature syndromes associated with accelerated bone maturation (Gibson and Briggs, 2016; Kim et al., 2020; Uchida et al., 2020; Wei et al., 2021). At present, only 65 missense/non-sense and 26 small deletion/insertion variants in the *ACAN* gene that are associated with short stature have been recorded in the Human Gene Mutation Database (HGMD; data retrieved in November 2021).

**FIGURE 2:**
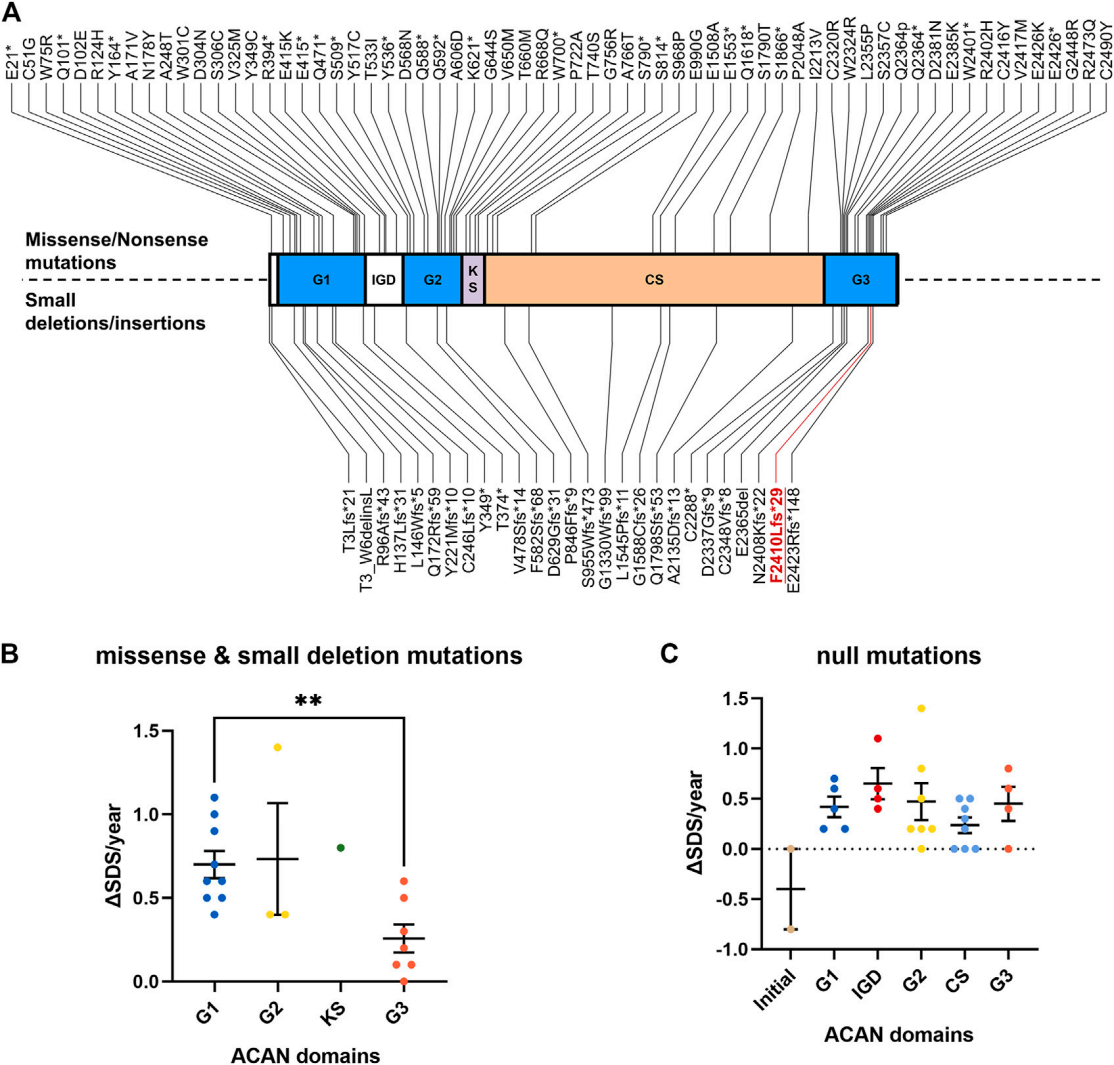
Overview of all known ACAN mutations. **(A)** The red font symbolizes the mutation identified in this study. **(B)** The increasing SDS for each year (ΔSDS/year) of patients with ACAN missense and small deletion mutations during GH treatment. **(C)** The increasing SDS for each year (ΔSDS/year) of patients with ACAN null mutations during GH treatment. G1, globular domain 1; IGD, interglobular domain; G2, globular domain 2; KS, keratin sulfate attachment domain; CS, chondroitin sulfate attachment domain; G3, globular domain 3; SDS, standard deviation scores. **p<0.01.

Aggrecan contains three globular domains (G1 and, G2 at the N-terminus and G3 at the C-terminal), one keratan sulfate domain, and one chondroitin sulfate domain. It also contains two epidermal growth factor-like motifs, one C-type lectin (CLD), and one complement regulatory protein-like motif in the G3 domain (Tompson et al., 2009; Liang et al., 2020). Previous studies have shown that CLD may mediate the interaction between aggrecan and tenascin in coordination with Ca^2+^ (Lundell et al., 2004; Diao and Tajkhorshid, 2008). Mutations of the *ACAN* gene (p.Phe2410Leufs*9), which was also located in the CLD domain of G3 (Figure 2A), may lead to the dysfunction of these interactions. Noticeably, Gkourogianni et al. also described an *ACAN* non-sense mutation (p.Trp2401*) that located in the CLD domain and led to short stature and early-onset osteoarthritis (Gkourogianni et al., 2017). Our study may provide additional evidence for the role of CLD in cartilage development.

In this study, we used WES to identify a novel p. Phe2410Leufs*9 frameshift mutation in exon 16 of *ACAN* in a family with short stature and advanced BA. Treatment with GH was performed since his age of 4 years and 7 months. However, the effectiveness of this treatment was limited during the first year of therapy (Figure 1C). Since GH facilitates growth by promoting the release of insulin-like growth factor 1, thus inducing chondrocyte proliferation and hypertrophy (Xu et al., 2018), treatment for a longer time and/or combination therapy may need to restore the normal ECM assembly and improve the height of the patient (van der Steen et al., 2017; Lin et al., 2021).

To clarify the relationship between *ACAN* mutations and the outcome of GH treatment, we reviewed all previously reported *ACAN* patients and their prognosis data during GH treatment (Table 1). A total of 49 *ACAN* patients was included. Most of these *ACAN* patients were firstly cued with different dose of GH (from 30 to 66 μg/kg/day) at age of 5–9. The increasing SDS for each year (ΔSDS/year) were calculated after their GH treatment. The range of ΔSDS/year was from −0.8 to 1.4 and the duration of GH treatment was from 1.5 months to 97 months. As we summarized, for missense and small deletion mutations, patients with *ACAN* mutations in G3 domain showed significant lower ΔSDS/year compared with other ACAN domains (Figure 2B). Especially the cases in Xu et al. (Xu et al., 2018) (Gln2364Pro) and Gkourogianni et al. (Gkourogianni et al., 2017) (Leu2355Pro), the ΔSDS/year were 0.0 or 0.3 even with a relative high dose GH (>40 μg/kg/day) and long duration time of treatment (91 or 18 months, respectively) (Table 1). When we compared the therapeutic differences between non-sense and frameshift mutations in each domain, most of these patients showed a similar GH sensitivity (Figure 2C). We also compared the ΔSDS/year data from who received GH combined with or without gonadotropin-releasing hormone analog (GnRHa). However, due to the limited cases, we found no difference. An example from Xu et al. (Xu et al., 2018) showed the ΔSDS/year only have a slight changed in the same *ACAN* mutation (Gln2364Pro) patients that treatment with or without GnRHa (Table 1). Which may suggest the little effect of GnRHa treatment in *ACAN* patients. However, much more clinical evidence is still demanded. All combined, the above information of point and null variants both suggesting G3, as the last functional domain of ACAN, may be critical in the development of short stature and GH treatment.

**TABLE 1:** Summarized of reported *ACAN* patients and their prognosis data during GH treatment.

ACAN domain	AA change	Sex	Age at Start GH	Height SDS at Start GH	Height SDS at End GH	ΔSDS	ΔSDS/year	Treat	PMID
—	(c.2T>C)	M	14.7	−4.1	−4.1	0.0	0.0	GH (49.5 μg/kg/d, 4.8 m)	36387899
—	(c.70 + 1G>A)	M	14.3	−4.9	−5.0	−0.1	−0.8	GH (43 μg/kg/d, 1.5 m)	35620465
G1	p.Ile73Serfs*12	F	7.0	−2.5	−2.1	0.4	0.4	GH (32.9 μg/kg/d, 12 m)	34653508
G1	p.Trp75Arg	M	5.5	−2.3	−1.3	1.0	1.0	GH (NA, 12 m)	27870580
G1	p.Trp75Arg	F	8.2	−1.6	−1.1	0.5	0.5	GH (NA, 12 m)	27870580
G1	p.Arg93Alafs*	M	8.6	−2.8	−2.6	0.2	0.2	GH (NA, 11 m) + AI (11 m)	27870580
G1	p.Arg93Alafs*	M	6.2	−2.0	−1.6	0.4	0.2	GH (NA, 30 m)	27870580
G1	p.Val94_Ile112del	M	3.4	−2.2	−3.3	1.1	1.1	GH (50–48 μg/kg/d, 12 m)	34922359
G1	p.Val94_Ile112del	M	5.0	−1.1	−1.8	0.7	0.7	GH (50–48 μg/kg/d, 12 m)	34922359
G1	p.Ala144Pro	M	8.2	−3.4	−2.9	0.5	0.6	GH (36.3 μg/kg/d, 9.6 m)	36387899
G1	p.Tyr164*	F	7.4	−3.1	−3.7	0.6	0.6	GH (50–48 μg/kg/d, 12 m)	34922359
G1	p.Ala180Thr	M	4.5	−2.0	−0.9	1.1	0.9	GH (53 μg/kg/d, 14 m)	35620465
G1	p.Trp203Cys	M	6.0	−4.3	−4.7	0.4	0.4	GH (50–48 μg/kg/d, 12 m)	34922359
G1	p.Val255_Glu352del	F	8.1	−3.0	−3.6	0.6	0.6	GH (50–48 μg/kg/d, 12 m)	34922359
G1	p.Thr282Ile	F	8.2	−3.8	−3.2	0.6	0.5	GH (33–39.6 μg/kg/d, 13.2 m)	36387899
G1	p.Trp301*	M	6.3	−2.9	−2.4	0.5	0.7	GH (33 μg/kg/d, 8.4 m)	36387899
IGD	p.Gly391Valfs*7	F	2.4	−2.6	−3.7	1.1	1.1	GH (50–48 μg/kg/d, 12 m)	34922359
IGD	p.Glu415*	F	8.5	−1.7	−0.8	0.9	0.4	GH (NA, 29 m) + GnRHa (13 m)	27870580
IGD	p.Val478Serfs*	M	7.4	−2.9	−1.4	1.5	0.5	GH (NA, 36 m)	27870580
IGD	(c.1429 + 1G>T)	F	6.0	−2.0	−1.4	0.6	0.6	GH (53 μg/kg/d, 12 m)	35620465
G2	p.Tyr517*	M	10.0	−3.0	−2.8	0.2	0.2	GH (28.6 μg/kg/d, 12 m)	34653508
G2	p.Tyr517*	F	7.2	−2.7	−2.5	0.2	0.2	GH (32.9 μg/kg/d, 12 m)	34653508
G2	p.Tyr536*	F	4.9	−3.7	−3.9	−0.2	0.0	GH (33 μg/kg/d, 97 m) + GnRHa (22 m) + GH (66 μg/kg/d, 12 m)	27710243
G2	p.Tyr536*	M	11.9	−2.4	−1.6	0.8	0.2	GH (66 μg/kg/d, 42 m) + GnRHa (20 m) + AI (10 m)	27710243
G2	p.Ala606Asp	F	7.0	−0.7	−0.1	0.6	0.4	GH (56.7 μg/kg/d, 18 m)	35620465
G2	p.Ala606Asp	M	2.9	−2.1	−1.4	0.7	1.4	GH (40 μg/kg/d, 6 m)	35620465
G2	p.Ala606Asp	M	9.4	−2.9	−1.9	1.0	0.4	GH (NA, 30 m)	33471655
G2	p.Arg675*	M	3.6	−2.5	−3.9	1.4	1.4	GH (50–48 μg/kg/d, 12 m)	34922359
G2	p.Arg675*	F	9.7	−3.5	−4.3	0.8	0.8	GH (50–48 μg/kg/d, 12 m)	34922359
G2	p.Arg675*	M	6.0	−3.8	−3.0	0.8	0.5	GH (56.1–62.7 μg/kg/d, 18 m)	36387899
KS	p.Gly756Arg	M	3.9	−2.9	−2.2	0.7	0.8	GH (NA, 11 m)	33471655
CS	p.Val1464*	F	10.8	−2.7	−2.6	0.1	0.0	GH (1.0–1.6 mg/d, 46.8 m) + GnRHa (14.4 m)	34456977
CS	p.Val1464*	F	8.2	−2.6	−2.6	0.0	0.0	GH (0.7–1.0 mg/d, 40.8 m)	34456977
CS	p.Gly1588fs	M	12.3	−2.7	−2.5	0.2	0.0	GH (33 μg/kg/d, 76 m) + GnRHa (26 m)	27710243
CS	p.Ile1686Metfs*13	M	12.8	−2.7	−2.6	0.1	0.2	GH (53 μg/kg/d, 5 m) + letrozole (2.5 mg qd)	35620465
CS	p.Leu 1815fs	M	4.8	−4.4	−3.1	1.3	0.5	GH (48 μg/kg/d, 30 m)	33606014
CS	p.Gly 1861fs	F	9.5	−2.9	−2.3	0.6	0.4	GH (48 μg/kg/d, 19 m)	33606014
CS	p.Pro2059Leufs*2	M	6.6	−3.3	−3.0	0.3	0.5	GH (56.1–62.7 μg/kg/d, 7.92 m)	36387899
CS			11.8	−2.5	−2.4	0.1	0.3	GH (49.5 μg/kg/d, 3.6 m)	36387899
G3	p.Leu2355Pro	F	12.0	−3.1	−3.0	0.1	0.1	AI (24 m) + GH (NA, 18 m)	27870580
G3	p.Gln2364Pro	M	6.4	−3.7	−3.8	0.0	0.0	GH (60 μg/kg/d, 91 m)	29769040
G3	p.Gln2364Pro	F	5.6	−2.1	−1.1	1.0	0.3	GH (40–50 μg/kg/d, 48 m) + GnRHa (30 m)	29769040
G3	p.Gln2364Pro	M	7.8	−0.9	−0.3	0.6	0.1	GH (50 μg/kg/d, 60 m) + GnRHa (8 m)	29769040
G3	p.Gln2364*	M	11.7	−2.7	−2.8	−0.1	0.0	GH (66 μg/kg/d, 66 m) + GnRHa (24 m) + AI (13 m) + AI (7 m)	27710243
G3	p.Trp2401*	F	5.2	−1.1	−1.5	0.4	0.4	GH (50–48 μg/kg/d, 12 m)	34922359
G3	p.Trp2401*	F	6.9	−2.3	−2.9	0.6	0.6	GH (50–48 μg/kg/d, 12 m)	34922359
G3	p.Phe2410Leufs*9	M	4.6	−2.6	−1.8	0.8	0.8	GH (50 μg/kg/d, 13 m)	This study
G3	p.Val2417Met	F	5.5	−1.2	−0.6	0.6	0.2	GH (NA, 44 m)	27870580
G3	p.Arg2489Gly	M	7.4	−2.1	−1.7	0.4	0.5	GH (43 μg/kg/d, 9 m)	35620465
G3	p.Cys2490Tyr	M	15.1	−4.5	−4.4	0.1	0.6	GH (NA, 2 m) + GnRHa (2 m)	33471655

G1, globular domain 1; IGD, interglobular domain; G2, globular domain 2; KS, keratin sulfate attachment domain; CS, chondroitin sulfate attachment domain; G3, globular domain 3; M, male; F, female; GH, growth hormone; SDS, standard deviation score; d, day; m, month(s); NA, not applicable; GnRHa, gonadotropin-releasing hormone analog; AI, aromatase inhibitor.

In summary, we report a novel mutation in *ACAN* (c.7230delT; p. Phe2410Leufs*9) in a Chinese family with short stature and advanced BA. Our analyses not only further confirmed the clinical diagnosis of short stature in the patients, but also expanded the mutation spectrum of *ACAN* and contributed to the genetic diagnosis and counseling of patients with short stature.

## Data Availability

The data that support the findings of this study are not publicly available because they contain information that could compromise the privacy of research participants; however, these data are available from the corresponding author upon reasonable request. The variation information has been submitted to ClinVar (SCV002819138).
