# Supplementary material for: Case report: A novel heterozygous frameshift mutation of ACAN in a Chinese family with short stature and advanced bone age
Source: Front Genet. 2023 Mar 21;14:1101695. doi: 10.3389/fgene.2023.1101695 (PMC10070732; doi:10.3389/fgene.2023.1101695)
Supplement: Supplementary file 1 [file Table1.DOCX]

Supplementary Material

# Supplementary Figure


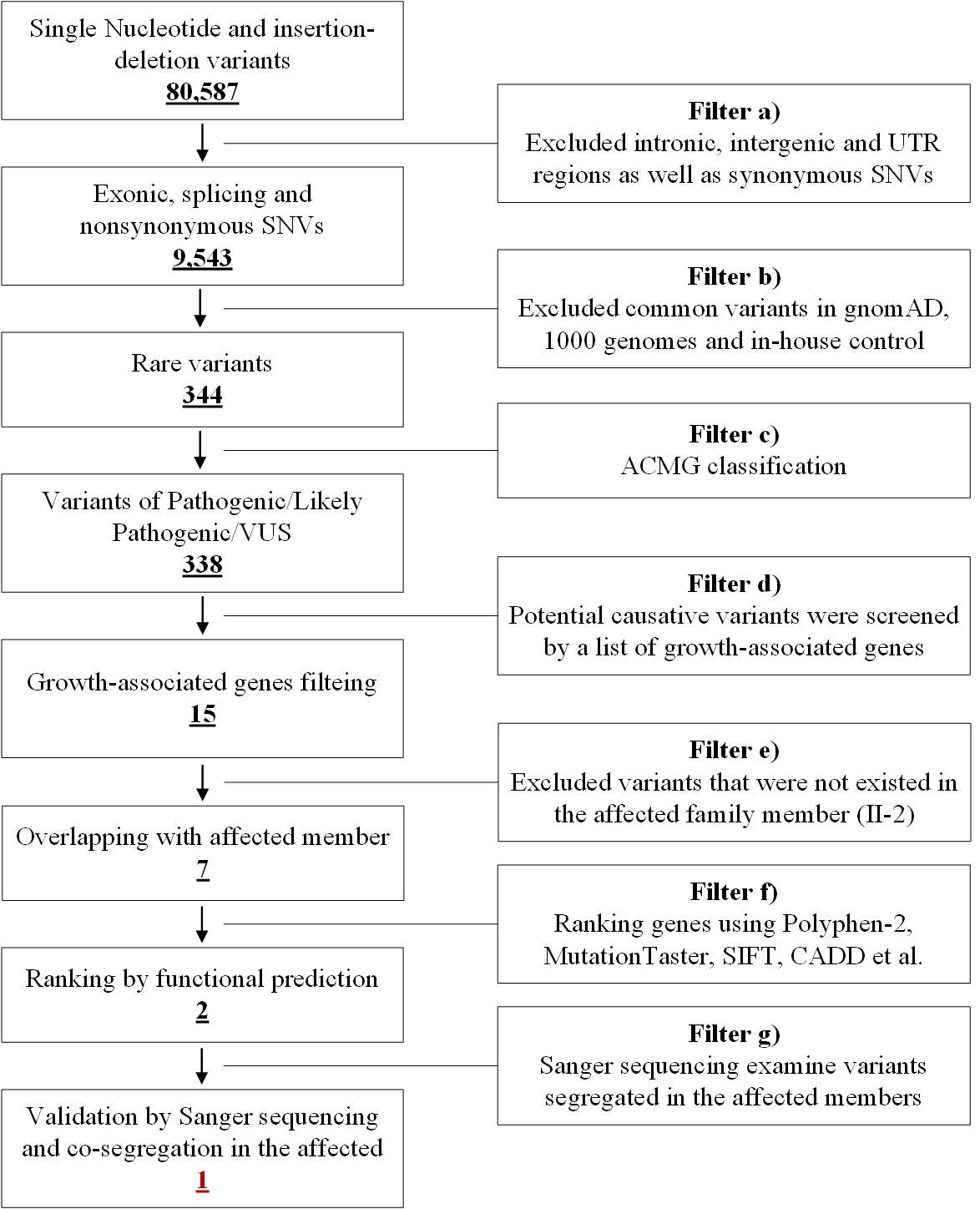


**Supplementary Figure 1.** Schematic representation of the filter strategies employed in the present study. UTR, untranslated region; SNV, single nucleotide variant; ACMG, American College of Medical Genetics; VUS, variant of unknown significance.

# Supplementary Table

**Supplementary Table 1. List of growth-associated genes**

| *AAAS* | *EFNB1* | *MCPH1* | *RNF213* |
| --- | --- | --- | --- |
| *AARS* | *EFTUD2* | *MCTP2* | *RNF216* |
| *AASS* | *EHHADH* | *MECP2* | *RNU4ATAC* |
| *ABCA12* | *EHMT1* | *MED12* | *ROGDI* |
| *ABCB11* | *EIF2AK3* | *MEF2C* | *ROR2* |
| *ABCB4* | *EIF4A3* | *MESP2* | *RPGRIP1L* |
| *ABCB6* | *ELAC2* | *MGAT2* | *RPL11* |
| *ABCB7* | *ELN* | *MGP* | *RPL15* |
| *ABCC8* | *ELOVL4* | *MIR17HG* | *RPL26* |
| *ABCD4* | *EMG1* | *MKKS* | *RPL35A* |
| *ABCG5* | *ENPP1* | *MKRN3* | *RPL5* |
| *ABCG8* | *EP300* | *MKS1* | *RPS10* |
| *ABHD5* | *EPB42* | *MLXIPL* | *RPS17* |
| *ACACA* | *EPCAM* | *MLYCD* | *RPS19* |
| *ACAN* | *EPG5* | *MMP13* | *RPS24* |
| *ACP5* | *EPHX1* | *MMP14* | *RPS26* |
| *ACTB* | *ERBB2* | *MMP2* | *RPS29* |
| *ACTG1* | *ERCC1* | *MMP9* | *RPS6KA3* |
| *ADAMTS10* | *ERCC2* | *MOCS1* | *RPS7* |
| *ADAMTS17* | *ERCC3* | *MOCS2* | *RSPH4A* |
| *ADAMTS2* | *ERCC4* | *MPL* | *RSPH9* |
| *ADAMTSL2* | *ERCC5* | *MPLKIP* | *RTEL1* |
| *ADNP* | *ERCC6* | *MPV17* | *RTTN* |
| *ADRB2* | *ERCC8* | *MPZ* | *RUNX2* |
| *ADSL* | *ESCO2* | *MRPS22* | *RYR1* |
| *AFF2* | *EVC* | *MT-CO1* | *SALL1* |
| *AGA* | *EVC2* | *MT-CO2* | *SALL4* |
| *AGK* | *EXOSC3* | *MT-CO3* | *SAR1B* |
| *AGL* | *EXT1* | *MT-CYB* | *SAT1* |
| *AGPS* | *EXT2* | *MTFMT* | *SATB2* |
| *AGRP* | *EYA1* | *MT-ND1* | *SBDS* |
| *AKT1* | *FAM111A* | *MT-ND5* | *SC5D* |
| *ALDH18A1* | *FAM111B* | *MT-ND6* | *SCN4A* |
| *ALDH3A2* | *FAM20C* | *MTR* | *SCN9A* |
| *ALDOA* | *FAM58A* | *MT-RNR1* | *SCO1* |
| *ALG1* | *FANCA* | *MT-TF* | *SCRIB* |
| *ALMS1* | *FANCB* | *MT-TK* | *SDCCAG8* |
| *ALOX12B* | *FANCC* | *MT-TL1* | *SDHA* |
| *ALOXE3* | *FANCD2* | *MT-TQ* | *SDHAF1* |
| *ALPL* | *FANCE* | *MT-TS1* | *SDHB* |
| *ALX3* | *FANCF* | *MT-TS2* | *SDHC* |
| *ALX4* | *FANCG* | *MT-TV* | *SDHD* |
| *AMER1* | *FANCI* | *MT-TW* | *SEC23A* |
| *AMPD2* | *FANCL* | *MVK* | *SECISBP2* |
| *ANK1* | *FANCM* | *MYCN* | *SEMA3E* |
| *ANKRD11* | *FAR1* | *MYH3* | *SEPN1* |
| *ANTXR1* | *FARS2* | *MYH7* | *9-Sep* |
| *AP1S1* | *FASTKD2* | *MYH8* | *SERPINF1* |
| *AP4B1* | *FAT4* | *MYO5B* | *SERPINH1* |
| *AP4E1* | *FBN1* | *NAA10* | *SETBP1* |
| *AP4S1* | *FBXL4* | *NALCN* | *SF3B4* |
| *APOPT1* | *FCGR2B* | *NBAS* | *SFXN4* |
| *AQP2* | *FERMT1* | *NBN* | *SGCE* |
| *ARG1* | *FGD1* | *NDE1* | *SH3PXD2B* |
| *ARHGAP26* | *FGF10* | *NDN* | *SHH* |
| *ARID1A* | *FGF17* | *NDST1* | *SHOC2* |
| *ARID1B* | *FGF23* | *NDUFA1* | *SHOX* |
| *ARL6* | *FGF8* | *NDUFA10* | *SHROOM4* |
| *ARSB* | *FGFR1* | *NDUFA11* | *SIL1* |
| *ARSE* | *FGFR2* | *NDUFA12* | *SIM1* |
| *ARVCF* | *FGFR3* | *NDUFA2* | *SIX6* |
| *ARX* | *FIG4* | *NDUFA9* | *SKIV2L* |
| *ASAH1* | *FKBP10* | *NDUFAF1* | *SLC10A2* |
| *ASCL1* | *FLNA* | *NDUFAF2* | *SLC12A1* |
| *ASL* | *FLNB* | *NDUFAF3* | *SLC16A1* |
| *ASPM* | *FLVCR2* | *NDUFAF4* | *SLC17A5* |
| *ASXL1* | *FOXE1* | *NDUFAF5* | *SLC19A2* |
| *ATM* | *FOXRED1* | *NDUFAF6* | *SLC20A2* |
| *ATP5A1* | *FSHR* | *NDUFB3* | *SLC25A13* |
| *ATP6V0A2* | *FTCD* | *NDUFB9* | *SLC26A2* |
| *ATP6V0A4* | *FTO* | *NDUFS1* | *SLC26A3* |
| *ATP6V1B1* | *FUCA1* | *NDUFS2* | *SLC26A4* |
| *ATP7A* | *G6PC* | *NDUFS3* | *SLC29A3* |
| *ATP8A2* | *G6PC3* | *NDUFS4* | *SLC2A2* |
| *ATP8B1* | *G6PD* | *NDUFS6* | *SLC34A1* |
| *ATPAF2* | *GALNS* | *NDUFS7* | *SLC34A3* |
| *ATR* | *GAN* | *NDUFS8* | *SLC35C1* |
| *ATRIP* | *GATA1* | *NDUFV1* | *SLC35D1* |
| *ATRX* | *GATA4* | *NDUFV2* | *SLC37A4* |
| *AUH* | *GATA6* | *NEU1* | *SLC39A13* |
| *AUTS2* | *GBA* | *NF1* | *SLC39A4* |
| *AVPR2* | *GCK* | *NFIX* | *SLC4A1* |
| *B3GALT6* | *GDF1* | *NHP2* | *SLC4A4* |
| *B3GALTL* | *GDF5* | *NIN* | *SLC5A2* |
| *B3GAT3* | *GDF6* | *NIPAL4* | *SLC5A5* |
| *B4GALT7* | *GDNF* | *NIPBL* | *SLC6A19* |
| *BANF1* | *GFM1* | *NKX2-1* | *SLC6A8* |
| *BBIP1* | *GH1* | *NKX2-5* | *SLC7A7* |
| *BBS1* | *GHR* | *NKX2-6* | *SLX4* |
| *BBS10* | *GHRHR* | *NKX3-2* | *SMAD4* |
| *BBS12* | *GHSR* | *NLRC4* | *SMARCA2* |
| *BBS2* | *GJA1* | *NLRP3* | *SMARCA4* |
| *BBS4* | *GJB2* | *NODAL* | *SMARCAL1* |
| *BBS5* | *GJB3* | *NOG* | *SMARCB1* |
| *BBS7* | *GJB4* | *NOP10* | *SMARCE1* |
| *BBS9* | *GJB6* | *NOTCH1* | *SMC1A* |
| *BCOR* | *GK* | *NOTCH2* | *SMC3* |
| *BCS1L* | *GLA* | *NOTCH3* | *SMOC1* |
| *BDNF* | *GLB1* | *NPHP1* | *SMPD1* |
| *BLM* | *GLE1* | *NPHP3* | *SMS* |
| *BMP1* | *GLI2* | *NPHP4* | *SNAI2* |
| *BMP15* | *GLI3* | *NPHS1* | *SNAP29* |
| *BMP2* | *GLIS3* | *NPR2* | *SNRPB* |
| *BMPER* | *GLYCTK* | *NR0B1* | *SNRPN* |
| *BMPR1A* | *GMPPA* | *NR0B2* | *SOS1* |
| *BMPR1B* | *GNA11* | *NR1I3* | *SOST* |
| *BRAF* | *GNAS* | *NR3C2* | *SOX11* |
| *BRCA1* | *GNPAT* | *NR5A1* | *SOX2* |
| *BRCA2* | *GNPTAB* | *NRAS* | *SOX3* |
| *BRCC3* | *GNPTG* | *NRTN* | *SOX9* |
| *BRF1* | *GNRH1* | *NSD1* | *SP7* |
| *BRIP1* | *GORAB* | *NSDHL* | *SPG20* |
| *BTK* | *GP1BB* | *NSUN2* | *SPINK5* |
| *BUB1* | *GPC6* | *NT5C2* | *SPINT2* |
| *BUB1B* | *GPD1* | *NUBPL* | *SPR* |
| *BUB3* | *GPR143* | *OBSL1* | *SPRTN* |
| *C10orf2* | *GRHL2* | *OCRL* | *SPTA1* |
| *C12orf57* | *GRIA3* | *OFD1* | *SRCAP* |
| *C15orf41* | *GRM1* | *OGDH* | *SRY* |
| *C5orf42* | *GSC* | *OPA3* | *STAC3* |
| *CA2* | *GTF2H5* | *ORC1* | *STAMBP* |
| *CA8* | *GTPBP3* | *ORC4* | *STAT1* |
| *CANT1* | *GUCY2D* | *ORC6* | *STAT3* |
| *CASC5* | *GUSB* | *OSTM1* | *STAT5B* |
| *CASK* | *GYS2* | *OTX2* | *STEAP3* |
| *CASP8* | *H19* | *PALB2* | *STIL* |
| *CASR* | *HADH* | *PAPSS2* | *STIM1* |
| *CAV1* | *HARS2* | *PAX1* | *STK11* |
| *CBL* | *HCCS* | *PAX2* | *STRA6* |
| *CC2D2A* | *HCFC1* | *PAX6* | *STS* |
| *CCBE1* | *HDAC4* | *PAX8* | *STT3B* |
| *CCDC28B* | *HDAC6* | *PCCA* | *STX16* |
| *CCDC8* | *HDAC8* | *PCCB* | *SUCLA2* |
| *CD96* | *HERC2* | *PCNA* | *SUCLG1* |
| *CDAN1* | *HES7* | *PCNT* | *SUMF1* |
| *CDC6* | *HESX1* | *PCYT1A* | *SURF1* |
| *CDH3* | *HFE* | *PDE11A* | *TAB2* |
| *CDK5RAP2* | *HIRA* | *PDE3A* | *TACO1* |
| *CDKN1C* | *HLA-DQB1* | *PDE4D* | *TAT* |
| *CDSN* | *HMGA2* | *PDE6D* | *TAZ* |
| *CDT1* | *HMGB3* | *PDE8B* | *TBC1D20* |
| *CENPE* | *HMOX1* | *PDGFB* | *TBCE* |
| *CENPJ* | *HNF1B* | *PDGFRB* | *TBX1* |
| *CEP135* | *HNF4A* | *PDX1* | *TBX15* |
| *CEP152* | *HOXD13* | *PET100* | *TBX22* |
| *CEP164* | *HPRT1* | *PEX1* | *TBX3* |
| *CEP290* | *HRAS* | *PEX10* | *TBX5* |
| *CEP57* | *HSD11B2* | *PEX11B* | *TBX6* |
| *CEP63* | *HSD17B4* | *PEX12* | *TCTN3* |
| *CERS3* | *HSPG2* | *PEX13* | *TECPR2* |
| *CFC1* | *HYAL1* | *PEX14* | *TERT* |
| *CHCHD10* | *HYLS1* | *PEX16* | *TFAP2A* |
| *CHD7* | *IARS2* | *PEX19* | *TG* |
| *CHRNA1* | *IBA57* | *PEX2* | *TGDS* |
| *CHRNB1* | *IDH1* | *PEX26* | *TGFB1* |
| *CHRND* | *IDS* | *PEX3* | *TGFB3* |
| *CHRNG* | *IDUA* | *PEX5* | *TGM1* |
| *CHST14* | *IFIH1* | *PEX6* | *THRA* |
| *CHST3* | *IFITM5* | *PEX7* | *THRB* |
| *CHSY1* | *IFT122* | *PGAP3* | *TINF2* |
| *CKAP2L* | *IFT140* | *PGK1* | *TMCO1* |
| *CLCN5* | *IFT172* | *PGM1* | *TMEM165* |
| *CLCN7* | *IFT27* | *PGM3* | *TMEM173* |
| *CLCNKB* | *IFT43* | *PHC1* | *TMEM216* |
| *CLMP* | *IFT80* | *PHEX* | *TMEM237* |
| *CLP1* | *IGBP1* | *PHF6* | *TMEM67* |
| *CLPP* | *IGF1* | *PHGDH* | *TMEM70* |
| *CNGB3* | *IGF1R* | *PHKA2* | *TNFRSF11A* |
| *CNTNAP2* | *IGF2* | *PHKB* | *TNFRSF11B* |
| *COG1* | *IGHMBP2* | *PHKG2* | *TNFSF11* |
| *COL10A1* | *IHH* | *PHOX2B* | *TNNI2* |
| *COL11A1* | *IKBKAP* | *PIEZO2* | *TNNT3* |
| *COL11A2* | *IKBKG* | *PIGL* | *TP53* |
| *COL1A1* | *IL21* | *PIGO* | *TP63* |
| *COL1A2* | *IL2RG* | *PIK3CA* | *TPM2* |
| *COL27A1* | *IMPAD1* | *PIK3R1* | *TPO* |
| *COL2A1* | *INPPL1* | *PITX1* | *TRAPPC11* |
| *COL3A1* | *INS* | *PITX2* | *TRAPPC2* |
| *COL5A1* | *INSR* | *PKLR* | *TREX1* |
| *COL5A2* | *INVS* | *PLEC* | *TRH* |
| *COL6A2* | *IQCB1* | *PLEKHM1* | *TRIM32* |
| *COL7A1* | *IYD* | *PLK4* | *TRIM37* |
| *COL9A1* | *JAG1* | *PLOD2* | *TRIP11* |
| *COL9A2* | *JAGN1* | *PLOD3* | *TRMT10A* |
| *COL9A3* | *KANSL1* | *PLP1* | *TRNT1* |
| *COLEC11* | *KAT6B* | *PMM2* | *TRPS1* |
| *COMP* | *KCNJ1* | *PNKP* | *TRPV4* |
| *COMT* | *KCNJ10* | *PNPLA1* | *TSFM* |
| *COX10* | *KCNJ11* | *PNPLA2* | *TSHB* |
| *COX14* | *KCNJ2* | *PNPLA6* | *TSHR* |
| *COX15* | *KDM5C* | *PNPT1* | *TSPYL1* |
| *COX20* | *KDM6A* | *POC1A* | *TTC21B* |
| *COX6B1* | *KIAA0196* | *POLE* | *TTC37* |
| *COX7B* | *KIAA1033* | *POLG* | *TTC7A* |
| *CREBBP* | *KIAA1279* | *POLR3A* | *TTC8* |
| *CRIPT* | *KIAA2022* | *POLR3B* | *TTI2* |
| *CRTAP* | *KIF11* | *POMC* | *TUBB4A* |
| *CTC1* | *KIF1A* | *PORCN* | *TUBGCP6* |
| *CTCF* | *KIF22* | *POU1F1* | *TWIST1* |
| *CTDP1* | *KIF2A* | *PPARG* | *TXNL4A* |
| *CTLA4* | *KIF5C* | *PPIB* | *UBR1* |
| *CTNNB1* | *KIF7* | *PPOX* | *UCP3* |
| *CTNS* | *KISS1R* | *PQBP1* | *UFD1L* |
| *CTSA* | *KIT* | *PRKAR1A* | *UQCC2* |
| *CTSK* | *KLF1* | *PRKDC* | *UQCC3* |
| *CUL4B* | *KLLN* | *PROP1* | *UROC1* |
| *CUL7* | *KMT2A* | *PRPS1* | *UROD* |
| *CYB5R3* | *KMT2D* | *PSAT1* | *UROS* |
| *CYP11B1* | *KRAS* | *PSMB8* | *USB1* |
| *CYP11B2* | *KRT14* | *PSMC3IP* | *USP9X* |
| *CYP19A1* | *KRT5* | *PSPH* | *VDR* |
| *CYP21A2* | *L1CAM* | *PTDSS1* | *VLDLR* |
| *CYP27B1* | *LAMTOR2* | *PTEN* | *VPS13A* |
| *CYP2R1* | *LARGE* | *PTF1A* | *VPS13B* |
| *CYP4F22* | *LARP7* | *PTH1R* | *VPS53* |
| *DCHS1* | *LARS2* | *PTHLH* | *WDPCP* |
| *DCX* | *LBR* | *PTPN11* | *WDR19* |
| *DDHD2* | *LEMD3* | *PTPN22* | *WDR34* |
| *DDR2* | *LEPR* | *PUF60* | *WDR35* |
| *DDX11* | *LEPRE1* | *PUS1* | *WDR60* |
| *DGUOK* | *LFNG* | *PYCR1* | *WDR62* |
| *DHCR24* | *LHX3* | *PYGL* | *WDR73* |
| *DHCR7* | *LHX4* | *RAB18* | *WDR81* |
| *DHODH* | *LIAS* | *RAB23* | *WFS1* |
| *DKC1* | *LIFR* | *RAB33B* | *WHSC1* |
| *DLL3* | *LINS* | *RAB3GAP1* | *WISP3* |
| *DLX5* | *LIPN* | *RAB3GAP2* | *WNT1* |
| *DMXL2* | *LMBR1* | *RAB40AL* | *WNT5A* |
| *DNA2* | *LMBRD1* | *RAD21* | *WNT7A* |
| *DNAJC19* | *LMNA* | *RAD50* | *WRAP53* |
| *DNAJC3* | *LMX1B* | *RAD51C* | *WRN* |
| *DNASE1* | *LRBA* | *RAF1* | *WT1* |
| *DNMT3B* | *LRP5* | *RAI1* | *WWOX* |
| *DOK7* | *LTBP2* | *RAPSN* | *XYLT1* |
| *DOLK* | *LTBP3* | *RARB* | *YARS2* |
| *DPP6* | *LZTFL1* | *RBBP8* | *ZBTB16* |
| *DPYD* | *MAF* | *RBCK1* | *ZBTB18* |
| *DPYS* | *MAGEL2* | *RBM10* | *ZBTB20* |
| *DSG1* | *MALT1* | *RBM28* | *ZBTB24* |
| *DST* | *MAN2B1* | *RBM8A* | *ZC4H2* |
| *DUOX2* | *MAP2K1* | *RDH11* | *ZDHHC15* |
| *DUOXA2* | *MAP2K2* | *RECQL4* | *ZEB2* |
| *DYM* | *MARS2* | *RET* | *ZFP57* |
| *DYNC1H1* | *MASP1* | *RFT1* | *ZFPM2* |
| *DYNC2H1* | *MATN3* | *RFX6* | *ZMPSTE24* |
| *EBP* | *MBD5* | *RIN2* | *ZMYND11* |
| *ECE1* | *MBTPS2* | *RIPK4* | *ZNF335* |
| *ECEL1* | *MC4R* | *RIT1* | *ZNF592* |
| *EDN3* | *MCM4* | *RNASEH2A* | *ZNF674* |
| *EDNRB* | *MCM9* | *RNF168* | *ZNF81* |

**Supplementary Table 2. Variants identified by WES in combination with growth-associated gene-filtering and affected member overlapping in the proband**

| **POS** | **Gene Name** | **Variant** | **OMIM** | **Consequence** | **ACMG** | **GnomAD_exome_AFpopmax** | **CADD_Phred** | **SIFT** | **MutationTaster** | **Polyphen2_HVAR** |
| --- | --- | --- | --- | --- | --- | --- | --- | --- | --- | --- |
| chr2: 120003224-120003224 | *STEAP3* | NM_018234.2:c.152G>A:p.Gly51Asp | AD:Anemia, hypochromic microcytic, with iron overload 2 | missense_variant | **VUS**  pm1 pm2 pp3 | 1.36×10^-3^ | 26.7 | D | D | D |
| chr3: 170732385-170732385 | *SLC2A2* | NM_000340.1:c.244G>A:p.Ala82Thr | AR:Fanconi-Bickel syndrome  AD:{Diabetes mellitus, noninsulin-dependent} | missense_variant | **VUS**  pm1 pm2 bp4 | 0 | 1.11 | T | B | N |
| chr3: 187003830-187003830 | *MASP1* | NM_139125.3:c.20A>G:p.Tyr7Cys | AR:3MC syndrome 1 | missense_variant | **VUS**  pm2 bp4 | 1.77×10^-5^ | 7.537 | T | B | N |
| chr5: 60198296-60198296 | *ERCC8* | NM_000082.3:c.590G>A:p.Arg197His | AR:Cockayne syndrome, type A  AR:UV-sensitive syndrome 2 | missense_variant | **VUS**  pm1 pm2 | 5.78×10^-5^ | 29.4 | T | D | D |
| chr6: 43014031-43014031 | *CUL7* | NM_014780.4:c.2603A>G:p.Asn868Ser | AR:3-M syndrome 1 | missense_variant | **VUS**  pm1 bp4 | 6.44×10^-3^ | 14.12 | T | B | N |
| chr9: 80030912-80030912 | *VPS13A* | NM_033305.2:c.9515C>T:p.Pro3172Leu | AR:Choreoacanthocytosis | missense_variant | **VUS**  pm2 | 2.94×10^-4^ | 25.3 | D | B | D |
| chr15: 89416148-89416148 | *ACAN* | NM_013227.3:c.7230delT:p.Phe2410Leufs*9 | AD:?Spondyloepiphyseal dysplasia, Kimberley type  AD:Short stature and advanced bone age, with or without early-onset osteoarthritis and/or osteochondritis dissecans  AR:Spondyloepimetaphyseal dysplasia, aggrecan type | frameshift_deletion | **Likely Pathogenic**  pvs1_moderate pm1 pm2 pp3 | 0 | - | - | D | - |

**Supplementary Table 3. List of variants in the filter strategy c**

| **No** | **Level** | **Evidence** | **GeneID** | **AAChange** |
| --- | --- | --- | --- | --- |
| 1 | VUS | pm2 bp4 | *PLCH2* | NM_001303012.1:exon21:c.3269G>A:p.R1090H |
| 2 | VUS | pm2 bp4 | *TNFRSF9* | NM_001561.5:exon9:c.731G>A:p.R244Q |
| 3 | VUS | pm1 pm2 | *UBR4* | NM_020765.2:exon19:c.2381C>T:p.A794V |
| 4 | VUS | pm1 pm2 pp3 | *DDOST* | NM_005216.4:exon7:c.797A>G:p.D266G |
| 5 | VUS | pm2 | *EIF4G3* | NM_001198801.1:exon14:c.1893C>A:p.F631L |
| 6 | VUS | pm1 pm2 bp4 | *MYOM3* | NM_152372.3:exon33:c.3905G>A:p.R1302Q |
| 7 | VUS | pm2 | *SRRM1* | NM_005839.3:exon14:c.1898G>A:p.R633Q |
| 8 | VUS | pm2 bp4 | *DCDC2B* | NM_001099434.1:exon7:c.767G>A:p.R256Q |
| 9 | VUS | pm2 bp4 | *MACF1* | NM_012090.5:exon43:c.6316C>T:p.R2106C |
| 10 | VUS | pm1 pm2 bp4 | *PPIE* | NM_001195007.1:exon10:c.885C>A:p.N295K |
| 11 | VUS | pm1 pm2 bp4 | *ERMAP* | NM_001017922.1:exon4:c.412A>G:p.T138A |
| 12 | VUS | pm2 pp3 | *PIGK* | NM_005482.2:exon3:c.178C>T:p.R60* |
| 13 | VUS | pm2 bp4 | *CLCA2* | NM_006536.6:exon14:c.2668C>T:p.P890S |
| 14 | VUS | pm1 pm2 | *CELSR2* | NM_001408.2:exon1:c.1594G>A:p.A532T |
| 15 | VUS | pm2 bp4 | *C2CD4D* | NM_001136003.1:exon2:c.331C>A:p.P111T |
| 16 | VUS | bp1 pm2 bp4 | *FLG* | NM_002016.1:exon3:c.8596G>A:p.D2866N |
| 17 | VUS | pm2 | *SMCP* | NM_030663.2:exon2:c.100T>C:p.C34R |
| 18 | VUS | pm2 | *PBXIP1* | NM_020524.3:exon2:c.8C>T:p.S3F |
| 19 | VUS | pm1 pm2 | *GBA* | NM_001005741.2:exon7:c.605G>A:p.R202Q |
| 20 | VUS | pm1 pm2 | *LRRC71* | NM_144702.2:exon14:c.1530G>C:p.K510N |
| 21 | VUS | pm2 | *FCRL3* | NM_052939.3:exon11:c.1816C>T:p.L606F |
| 22 | VUS | pm1 pm2 pp3 | *ITLN1* | NM_017625.2:exon5:c.454G>A:p.V152M |
| 23 | VUS | pm2 | *PRRC2C* | NM_015172.3:exon8:c.930C>A:p.N310K |
| 24 | VUS | pm1 pm2 | *HMCN1* | NM_031935.2:exon51:c.7955C>T:p.A2652V |
| 25 | VUS | pm1 pm2 | *KDM5B* | NM_001314042.1:exon22:c.3203G>A:p.R1068H |
| 26 | VUS | bp4 | *LAX1* | NM_017773.3:exon4:c.332T>C:p.M111T |
| 27 | VUS | pm1 bp4 | *SLC26A9* | NM_052934.3:exon16:c.1733G>A:p.R578K |
| 28 | VUS | pm1 pm2 pp3 | *LAMB3* | NM_000228.2:exon6:c.479T>G:p.V160G |
| 29 | VUS | bp4 | *HLX* | NM_021958.3:exon1:c.451G>A:p.A151T |
| 30 | VUS | pm2 bp4 | *MRPL55* | NM_181462.2:exon4:c.119C>T:p.T40M |
| 31 | VUS | pm1 pm2 | *OBSCN* | NM_001098623.2:exon27:c.7345G>A:p.V2449M |
| 32 | VUS | pm2 pp3 | *ARID4B* | NM_016374.5:exon20:c.2639A>G:p.N880S |
| 33 | VUS | pm1 pm2 bp4 | *OR2L8* | NM_001001963.1:exon1:c.400C>T:p.R134C |
| 34 | VUS | pm2 bp4 | *FAM110C* | NM_001077710.2:exon1:c.448G>A:p.E150K |
| 35 | VUS | pm1 pm2 | *XDH* | NM_000379.3:exon22:c.2359C>T:p.R787W |
| 36 | VUS | pm1 pm2 | *MAP4K3* | NM_003618.3:exon26:c.1949A>G:p.K650R |
| 37 | VUS | pm2 | *ABCG5* | NM_022436.2:exon8:c.1068C>G:p.F356L |
| 38 | VUS | pm1 pm2 | *CLHC1* | NM_001135598.1:exon4:c.292C>T:p.R98W |
| 39 | VUS | pm1 | *DQX1* | NM_133637.2:exon7:c.1247T>C:p.L416P |
| 40 | VUS | pm2 | *TRABD2A* | NM_001277053.1:exon5:c.1054C>T:p.H352Y |
| 41 | VUS | pm2 | *TCF7L1* | NM_031283.2:exon12:c.1681T>G:p.S561A |
| 42 | VUS | pm2 bp4 | *VWA3B* | NM_144992.4:exon10:c.1444G>A:p.A482T |
| 43 | VUS | pm2 | *LIPT1* | NM_015929.3:exon3:c.71T>A:p.F24Y |
| 44 | VUS | pm1 pm2 pp3 | *TBC1D8* | NM_001102426.2:exon11:c.1813A>C:p.K605Q |
| 45 | VUS | pm1 pm2 pp3 | *STEAP3* | NM_018234.2:exon3:c.152G>A:p.G51D |
| 46 | VUS | pm1 pm2 bp4 | *LCT* | NM_002299.3:exon1:c.571C>G:p.Q191E |
| 47 | VUS | pm2 | *KYNU* | NM_003937.2:exon2:c.58C>T:p.L20F |
| 48 | VUS | pm1 pm2 pp3 | *KYNU* | NM_003937.2:exon13:c.1268T>C:p.V423A |
| 49 | VUS | pm1 bp4 | *GTDC1* | NM_001284238.1:exon14:c.1232A>G:p.H411R |
| 50 | VUS | pm2 bp4 | *TANK* | NM_001199135.1:exon4:c.296T>C:p.I99T |
| 51 | VUS | pm1 pm2 | *HAT1* | NM_003642.3:exon6:c.547A>T:p.M183L |
| 52 | VUS | pm1 pm2 | *TTN* | NM_001267550.2:exon358:c.105436G>A:p.A35146T |
| 53 | VUS | pm1 pm2 | *TTN* | NM_001267550.2:exon358:c.101708G>A:p.R33903H |
| 54 | VUS | pm2 bp4 | *TTN* | NM_001267550.2:exon28:c.6040A>G:p.T2014A |
| 55 | VUS | pm2 bp6 bp4 | *TTN* | NM_001267550.2:exon14:c.2230G>A:p.A744T |
| 56 | VUS | pm2 | *SLC11A1* | NM_000578.3:exon8:c.644T>C:p.V215A |
| 57 | VUS | pm1 pm2 | *ZNF142* | NM_001105537.2:exon8:c.4042C>T:p.R1348C |
| 58 | VUS | pm1 pm2 bp4 | *STK36* | NM_015690.4:exon3:c.145C>A:p.Q49K |
| 59 | VUS | pm2 bp6 pp3 | *WNT10A* | NM_025216.2:exon4:c.841C>G:p.P281A |
| 60 | VUS | pm1 pm2 pp3 | *EPHA4* | NM_004438.4:exon15:c.2647C>T:p.R883C |
| 61 | VUS | pm2 bp4 | *SPHKAP* | NM_001142644.1:exon7:c.2185C>T:p.R729W |
| 62 | VUS | pm1 pm2 | *SNED1* | NM_001080437.1:exon10:c.1408G>C:p.D470H |
| 63 | VUS | pm1 pm2 pp3 | *CNTN4* | NM_175607.2:exon15:c.1492A>G:p.T498A |
| 64 | VUS | pm2 | *EDEM1* | NM_014674.2:exon2:c.523A>G:p.I175V |
| 65 | VUS | pp3 | *KAT2B* | NM_003884.4:exon9:c.1369G>T:p.V457F |
| 66 | VUS | pm1 pm2 | *CLASP2* | NM_001207044.1:exon31:c.3352A>G:p.I1118V |
| 67 | VUS | pm1 pm2 | *CLASP2* | NM_001207044.1:exon12:c.1091G>A:p.S364N |
| 68 | VUS | pm2 bp4 | *EPM2AIP1* | NM_014805.3:exon1:c.1615A>G:p.I539V |
| 69 | VUS | pm1 pm2 bp4 | *DLEC1* | NM_007337.3:exon7:c.1249G>T:p.A417S |
| 70 | VUS | pm1 pp3 | *SCN11A* | NM_014139.2:exon6:c.763A>C:p.N255H |
| 71 | VUS |  | *PTPN23* | NM_015466.3:exon20:c.3298C>T:p.R1100C |
| 72 | VUS | pm1 pm2 pp3 | *PFKFB4* | NM_004567.3:exon8:c.698A>G:p.H233R |
| 73 | VUS | pm1 pm2 pp3 | *WDR6* | NM_018031.4:exon2:c.2414C>T:p.S805F |
| 74 | VUS | pm1 pm2 pp3 | *CYB561D2* | NM_007022.4:exon3:c.140G>A:p.W47* |
| 75 | VUS | pm1 pm2 pp3 | *SEMA3G* | NM_020163.2:exon7:c.796G>A:p.V266M |
| 76 | VUS | pm1 pm2 pp3 | *GBE1* | NM_000158.3:exon6:c.773C>T:p.A258V |
| 77 | VUS |  | *TRAT1* | NM_016388.3:exon6:c.536G>T:p.R179L |
| 78 | VUS | pm2 bp4 | *B3GALNT1* | NM_003781.3:exon5:c.23T>C:p.V8A |
| 79 | VUS | pm1 pm2 bp4 | *SLC2A2* | NM_000340.1:exon3:c.244G>A:p.A82T |
| 80 | VUS | pm2 pp3 | *EIF4G1* | NM_198241.2:exon3:c.53T>C:p.L18P |
| 81 | VUS | pm2 bp4 | *MASP1* | NM_139125.3:exon2:c.20A>G:p.Y7C |
| 82 | VUS |  | *HRASLS* | NM_020386.4:exon1:c.121C>T:p.R41C |
| 83 | VUS | pm2 bp4 | *MUC4* | NM_018406.6:exon2:c.11921G>T:p.R3974L |
| 84 | VUS | pm2 bp4 | *MUC4* | NM_018406.6:exon2:c.9226A>T:p.T3076S |
| 85 | VUS | pm2 pm3_supporting | *MUC4* | . |
| 86 | VUS | pm2 pm3_supporting | *MUC4* | . |
| 87 | VUS | pm2 pm3_supporting | *MUC4* | . |
| 88 | VUS | pm2 pp3 | *CLRN2* | NM_001079827.2:exon1:c.236G>T:p.R79L |
| 89 | VUS | pm1 pm2 bp4 | *CENPC* | NM_001812.2:exon8:c.887C>A:p.T296K |
| 90 | VUS | pm1 pm2 pp3 | *NFKB1* | NM_003998.3:exon22:c.2538T>G:p.N846K |
| 91 | VUS | pm1 pm2 | *LRIT3* | NM_198506.4:exon3:c.773C>T:p.T258I |
| 92 | VUS | pm1 pm2 pp3 | *ETFDH* | NM_004453.3:exon11:c.1430T>C:p.I477T |
| 93 | VUS | pm2 | *ANP32C* | NM_012403.1:exon1:c.145A>C:p.K49Q |
| 94 | VUS | pm1 | *F11* | NM_000128.3:exon15:c.1843G>A:p.V615M |
| 95 | VUS | pm1 pm2 pp3 | *FAT1* | NM_005245.3:exon6:c.4117G>A:p.V1373I |
| 96 | VUS | pm1 pm2 | *TRIML1* | NM_178556.4:exon1:c.209G>A:p.R70H |
| 97 | VUS | pm2 | *C5orf38* | NM_178569.3:exon2:c.310C>T:p.P104S |
| 98 | VUS | pm2 | *ROPN1L* | NM_031916.4:exon5:c.685G>A:p.G229S |
| 99 | VUS | pm1 pm2 bp4 | *TTC23L* | NM_144725.3:exon5:c.388G>T:p.V130F |
| 100 | VUS |  | *C5orf42* | NM_023073.3:exon34:c.6905C>T:p.T2302M |
| 101 | VUS | pm2 bp4 | *SNX18* | NM_001102575.1:exon1:c.430T>C:p.Y144H |
| 102 | VUS | pm1 pm2 | *GZMA* | NM_006144.3:exon4:c.604C>T:p.R202* |
| 103 | VUS | pm1 pm2 | *ERCC8* | NM_000082.3:exon7:c.590G>A:p.R197H |
| 104 | VUS |  | *C5orf64* | NM_173667.3:exon3:c.109C>T:p.R37C |
| 105 | VUS | pm2 pp3 | *LIX1* | NM_153234.4:exon3:c.263C>T:p.A88V |
| 106 | VUS | pm1 pm2 pp3 | *CAMK4* | NM_001744.5:exon3:c.283C>T:p.R95C |
| 107 | VUS | pm2 pp3 | *MCC* | NM_001085377.1:exon5:c.815T>C:p.I272T |
| 108 | VUS |  | *BRD8* | NM_139199.1:exon23:c.3200G>T:p.G1067V |
| 109 | VUS | pm1 pm2 | *PCDHB14* | NM_018934.3:exon1:c.403G>A:p.D135N |
| 110 | VUS | pm1 pm2 | *PPP2R2B* | NM_181678.2:exon8:c.632C>T:p.T211M |
| 111 | VUS | pm1 | *TIGD6* | NM_030953.3:exon2:c.895C>A:p.Q299K |
| 112 | VUS | pm1 bp4 | *OR2V2* | NM_206880.1:exon1:c.189C>A:p.F63L |
| 113 | VUS | pm1 bp4 | *OR2V2* | NM_206880.1:exon1:c.687G>A:p.M229I |
| 114 | VUS | pm2 | *MYLK4* | NM_001012418.4:exon4:c.271C>A:p.R91S |
| 115 | VUS | pm1 pp3 | *SERPINB9* | NM_004155.5:exon7:c.985G>T:p.A329S |
| 116 | VUS | pm1 pm2 bp4 | *ANKS1A* | NM_015245.2:exon15:c.2362G>A:p.V788I |
| 117 | VUS | pm1 pm2 pp3 | *FKBP5* | NM_004117.3:exon9:c.895G>C:p.E299Q |
| 118 | VUS | pm2 bp4 | *C6orf132* | NM_001164446.2:exon4:c.1361C>T:p.A454V |
| 119 | VUS | pm1 bp4 | *CUL7* | NM_014780.4:exon12:c.2603A>G:p.N868S |
| 120 | VUS | pm1 pm2 | *RHAG* | NM_000324.2:exon4:c.572G>A:p.R191Q |
| 121 | VUS | pm2 | *RIMS1* | NM_014989.5:exon1:c.28C>T:p.P10S |
| 122 | VUS | pm1 pm2 bp4 | *CD109* | NM_133493.4:exon30:c.3823A>G:p.I1275V |
| 123 | VUS | pm2 | *FILIP1* | NM_015687.4:exon6:c.3553G>A:p.E1185K |
| 124 | VUS | pm2 | *DOPEY1* | NM_001199942.1:exon31:c.6134A>G:p.Y2045C |
| 125 | VUS |  | *CASP8AP2* | NM_001137668.1:exon7:c.2372T>G:p.I791R |
| 126 | VUS | pm2 bp4 | *GJA10* | NM_032602.1:exon1:c.1181C>T:p.P394L |
| 127 | VUS | pm1 pm2 | *AIM1* | NM_001624.3:exon14:c.4294T>G:p.F1432V |
| 128 | VUS | pm2 | *ARMC2* | NM_001286609.1:exon4:c.14C>G:p.S5C |
| 129 | VUS | pm2 | *RSPO3* | NM_032784.4:exon4:c.632G>A:p.R211Q |
| 130 | VUS | pm1 pm2 | *TAAR1* | NM_138327.2:exon1:c.500G>A:p.G167D |
| 131 | VUS | pm2 bp4 | *AKAP12* | NM_005100.3:exon4:c.4187C>T:p.P1396L |
| 132 | VUS | pp3 | *SYNE1* | NM_033071.3:exon88:c.16688T>C:p.M5563T |
| 133 | VUS | pm1 bp4 | *KIF25* | NM_030615.2:exon6:c.582T>A:p.D194E |
| 134 | VUS | pm2 bp4 | *C7orf72* | NM_001161834.2:exon2:c.640G>T:p.A214S |
| 135 | Benign | pm1 ba1 pp3 | *PSPH* | NM_004577.3:exon5:c.268G>A:p.G90S |
| 136 | VUS | pm1 bp4 | *MOSPD3* | NM_023948.4:exon3:c.437G>A:p.G146E |
| 137 | VUS | pm2 | *TFR2* | NM_003227.3:exon2:c.73C>T:p.R25C |
| 138 | VUS | pm1 pm2 pp3 | *MYL10* | NM_138403.4:exon8:c.622G>A:p.G208S |
| 139 | VUS | pm1 pm2 bp4 | *ADCK2* | NM_052853.3:exon1:c.662C>T:p.T221I |
| 140 | VUS | pm2 bp4 | *KEL* | NM_000420.2:exon2:c.67C>T:p.L23F |
| 141 | VUS | pm1 | *SSPO* | UNKNOWN |
| 142 | VUS | pm1 pm2 bp4 | *GIMAP7* | NM_153236.3:exon2:c.218G>A:p.S73N |
| 143 | VUS | pm1 pm2 bp4 | *GIMAP1-GIMAP5;GIMAP5* | NM_001199577.1:exon6:c.806C>T:p.T269M |
| 144 | VUS | pm1 pm2 bp4 | *KBTBD11* | NM_014867.2:exon2:c.1188G>C:p.R396S |
| 145 | VUS | pp3 | *PRAG1* | NM_001080826.2:exon2:c.739G>T:p.D247Y |
| 146 | VUS | pm2 | *RP1L1* | NM_178857.5:exon4:c.4700G>A:p.R1567H |
| 147 | VUS | bp4 | *RP1L1* | NM_178857.5:exon4:c.3971A>G:p.E1324G |
| 148 | VUS | pm1 pm2 | *PDGFRL* | NM_006207.2:exon3:c.280C>A:p.L94I |
| 149 | VUS | pm1 pm2 pp3 | *SLC18A1* | NM_003053.3:exon10:c.923C>T:p.S308F |
| 150 | VUS | pm2 | *FZD3* | NM_017412.3:exon8:c.1847G>C:p.R616P |
| 151 | VUS | pm1 pm2 | *SNTG1* | NM_018967.4:exon16:c.1060C>T:p.R354W |
| 152 | VUS | pm1 | *NSMAF* | NM_001144772.1:exon30:c.2668G>A:p.V890I |
| 153 | VUS | pm1 pm2 pp3 | *NSMAF* | NM_001144772.1:exon26:c.2219C>T:p.T740M |
| 154 | VUS |  | *C8orf87* | NM_001242668.1:exon1:c.5G>T:p.R2L |
| 155 | VUS | pm1 pm2 | *AZIN1* | NM_148174.3:exon10:c.932C>T:p.A311V |
| 156 | VUS | pm2 bp4 | *RNF139* | NM_007218.3:exon2:c.1949A>G:p.Q650R |
| 157 | VUS | pm2 bp4 | *GLI4* | NM_138465.3:exon2:c.41C>T:p.P14L |
| 158 | VUS | pm2 pp3 | *KIAA2026* | NM_001017969.2:exon4:c.2246A>C:p.K749T |
| 159 | VUS | pm2 | *TOPORS* | NM_005802.4:exon3:c.1855C>T:p.H619Y |
| 160 | VUS | pm2 bp4 | *KIF24* | NM_194313.2:exon11:c.2698A>G:p.N900D |
| 161 | VUS | pm2 bp4 | *TRPM3* | NM_020952.4:exon23:c.3045C>A:p.H1015Q |
| 162 | VUS | pm2 | *VPS13A* | NM_033305.2:exon72:c.9515C>T:p.P3172L |
| 163 | VUS | pm2 bp4 | *WHRN* | NM_015404.3:exon6:c.1349G>A:p.R450H |
| 164 | VUS | pm2 bp4 | *PPP1R26* | NM_014811.3:exon4:c.2654C>T:p.P885L |
| 165 | VUS | pm1 pm2 bp4 | *NOXA1* | NM_006647.1:exon2:c.206C>A:p.T69N |
| 166 | VUS | pm2 bp4 | *APBB1IP* | NM_019043.3:exon15:c.1657T>A:p.F553I |
| 167 | VUS | pm1 pm2 | *SVIL* | NM_003174.3:exon26:c.3774A>T:p.E1258D |
| 168 | Likely benign | bp6 bp4 | *BMS1* | NM_014753.3:exon10:c.1645G>A:p.A549T |
| 169 | VUS | pp3 | *NCOA4* | NM_001145260.1:exon9:c.829C>T:p.L277F |
| 170 | VUS | pm1 pm2 | *HERC4* | NM_022079.2:exon26:c.3037G>A:p.G1013R |
| 171 | VUS | pm1 pm2 pp3 | *HK1* | NM_000188.2:exon5:c.587G>A:p.R196Q |
| 172 | Likely pathogenic | pm1 pm2 ps1 pp3 | *CDH23* | NM_022124.5:exon27:c.3178C>T:p.R1060W |
| 173 | VUS | pm1 pm2 bp4 | *CDH23* | NM_022124.5:exon56:c.8029C>G:p.Q2677E |
| 174 | VUS | pm1 pm2 | *DNMBP* | NM_015221.3:exon5:c.2367A>G:p.I789M |
| 175 | VUS | pm2 | *CHUK* | NM_001278.4:exon10:c.1059A>C:p.E353D |
| 176 | VUS | pm2 | *PDZD7* | NM_001195263.1:exon9:c.1418A>T:p.K473M |
| 177 | VUS | pm2 | *CALHM3* | NM_001129742.1:exon2:c.527T>C:p.L176P |
| 178 | VUS | pm2 pp3 | *ADD3* | NM_016824.4:exon11:c.1474G>T:p.V492F |
| 179 | Benign | ba1 bp4 | *MUC6* | NM_005961.2:exon31:c.4957A>G:p.I1653V |
| 180 | VUS | bp6 | *MUC5B* | NM_002458.2:exon30:c.3926C>T:p.T1309M |
| 181 | VUS | bp4 | *MUC5B* | NM_002458.2:exon31:c.7253C>T:p.P2418L |
| 182 | VUS | pm1 pm2 bp4 | *OR52N1* | NM_001001913.1:exon1:c.592G>A:p.A198T |
| 183 | VUS | pm2 | *DNHD1* | NM_144666.2:exon21:c.4808G>A:p.R1603H |
| 184 | VUS | pm1 pm2 pp3 | *TUB* | NM_177972.2:exon3:c.164G>A:p.R55Q |
| 185 | VUS | pm2 bp4 | *SLC1A2* | NM_004171.3:exon5:c.661G>A:p.E221K |
| 186 | VUS | bp4 | *SYT13* | NM_020826.2:exon1:c.125G>A:p.R42Q |
| 187 | VUS | pm2 pp3 | *FNBP4* | NM_015308.3:exon8:c.1418G>T:p.S473I |
| 188 | VUS | pm1 pm2 bp4 | *LRRC55* | NM_001005210.2:exon1:c.192C>G:p.I64M |
| 189 | VUS | pm2 | *SART1* | NM_005146.4:exon11:c.1325G>A:p.R442Q |
| 190 | VUS | pm2 bp4 | *SPTBN2* | NM_006946.2:exon33:c.6491C>G:p.P2164R |
| 191 | VUS | pm2 | *MRGPRF* | NM_145015.4:exon3:c.89G>A:p.R30Q |
| 192 | VUS | pm2 | *PPFIA1* | NM_003626.3:exon17:c.2257C>T:p.R753W |
| 193 | VUS | pm2 | *NUMA1* | NM_006185.3:exon25:c.6067C>T:p.R2023C |
| 194 | VUS | pm1 pm2 | *FAT3* | NM_001008781.2:exon14:c.9877A>G:p.I3293V |
| 195 | VUS | bp4 | *CEP295* | NM_033395.1:exon15:c.4802A>G:p.D1601G |
| 196 | VUS | pvs1_moderate pm2 | *EXPH5* | NM_015065.2:exon6:c.3997C>T:p.R1333* |
| 197 | VUS | pm1 bp4 | *ARHGAP20* | NM_020809.3:exon16:c.1945G>A:p.V649I |
| 198 | VUS | pm1 pm2 pp3 | *VWF* | NM_000552.4:exon16:c.1958C>T:p.P653L |
| 199 | VUS | bp4 | *LPAR5* | NM_001142961.1:exon2:c.1006G>A:p.A336T |
| 200 | VUS | pm1 pm2 pp3 | *TPI1* | NM_001159287.1:exon1:c.221A>T:p.D74V |
| 201 | VUS_FP | pm2 | *DDX12P* | . |
| 202 | VUS | pm1 | *KLRF2* | NM_001190765.1:exon6:c.590C>T:p.A197V |
| 203 | VUS | pm2 bp4 | *TAS2R14* | NM_023922.1:exon1:c.352A>G:p.I118V |
| 204 | VUS | bp4 | *TAS2R19* | NM_176888.2:exon1:c.755T>C:p.L252P |
| 205 | VUS | bp4 | *TAS2R30* | NM_001097643.1:exon1:c.98T>C:p.I33T |
| 206 | VUS | bp4 | *TAS2R30* | NM_001097643.1:exon1:c.47T>C:p.I16T |
| 207 | VUS | bp4 | *TAS2R30* | NM_001097643.1:exon1:c.37A>G:p.I13V |
| 208 | VUS | pm2 bp4 | *PRB4* | NM_002723.5:exon3:c.668A>G:p.K223R |
| 209 | VUS | pm2 | *DDX11* | NM_030653.3:exon14:c.1420G>A:p.E474K |
| 210 | VUS | pm1 pm2 | *HDAC7* | NM_015401.4:exon15:c.1900A>T:p.S634C |
| 211 | VUS | pm2 | *SP1* | NM_138473.2:exon4:c.1786G>A:p.G596S |
| 212 | VUS | pm1 pm2 | *CALCOCO1* | NM_020898.2:exon4:c.343C>A:p.P115T |
| 213 | VUS | pm2 | *NACA* | NM_001113203.2:exon3:c.403G>A:p.A135T |
| 214 | VUS | pm1 pm2 pp3 | *LRP1* | NM_002332.2:exon15:c.2437G>C:p.G813R |
| 215 | VUS | pm1 pm2 bp4 | *MARS* | NM_004990.3:exon3:c.238C>T:p.L80F |
| 216 | VUS | pm1 pm2 | *LRIG3* | NM_153377.4:exon9:c.1117T>C:p.W373R |
| 217 | VUS | pm2 | *PTPRR* | NM_002849.3:exon6:c.784G>A:p.D262N |
| 218 | VUS | pm1 pm2 bp4 | *TRHDE* | NM_013381.2:exon16:c.2627T>A:p.F876Y |
| 219 | VUS | pm2 bp4 | *CCDC38* | NM_182496.2:exon10:c.895C>T:p.R299C |
| 220 | VUS | pm1 pm2 | *GAS2L3* | NM_174942.2:exon6:c.422A>T:p.Y141F |
| 221 | VUS | pm2 bp4 | *TDG* | NM_003211.4:exon9:c.1081A>G:p.N361D |
| 222 | VUS | pm2 | *SSH1* | NM_018984.3:exon3:c.177A>T:p.Q59H |
| 223 | VUS | pm1 | *GCN1* | NM_006836.1:exon49:c.6595C>T:p.R2199C |
| 224 | VUS | pm1 pm2 bp4 | *LRRC43* | NM_001098519.1:exon10:c.1624T>C:p.W542R |
| 225 | VUS | pm2 bp4 | *ANKLE2* | NM_015114.2:exon11:c.2306C>T:p.A769V |
| 226 | VUS | pm2 bp4 | *ANKLE2* | NM_015114.2:exon11:c.2305G>A:p.A769T |
| 227 | VUS | pm1 pm2 pp3 | *MIPEP* | NM_005932.3:exon12:c.1271A>C:p.H424P |
| 228 | VUS | pvs1_moderate pm2 pp3 | *DGKH* | NM_152910.5:NA:c.3214-2A>G:. |
| 229 | VUS | pm1 pm2 bp4 | *KCTD12* | NM_138444.3:exon1:c.187A>G:p.M63V |
| 230 | VUS | pm1 pm2 bp4 | *RNF31* | NM_017999.4:exon8:c.1465C>G:p.L489V |
| 231 | VUS | pm2 | *HECTD1* | NM_015382.3:exon25:c.4559C>T:p.S1520F |
| 232 | VUS | pm1 pm2 | *FRMD6* | NM_001267046.1:exon14:c.1664T>C:p.F555S |
| 233 | VUS | pm1 pm2 bp4 | *SYNE2* | NM_182914.2:exon55:c.11030A>G:p.N3677S |
| 234 | VUS | pm1 pm2 bp4 | *ADAM21* | NM_003813.3:exon2:c.1713T>G:p.I571M |
| 235 | VUS | pm1 pm2 bp4 | *SLC24A4* | NM_153648.3:exon17:c.1561C>T:p.R521W |
| 236 | VUS | pp2 pm2 | *DYNC1H1* | NM_001376.4:exon62:c.11641A>G:p.I3881V |
| 237 | VUS | pm2 | *MGA* | NM_001164273.1:exon17:c.6981G>T:p.Q2327H |
| 238 | VUS | pm2 bp4 | *ZNF106* | NM_022473.2:exon2:c.1501C>T:p.P501S |
| 239 | VUS | bp4 | *MAP1A* | NM_002373.5:exon4:c.4798T>A:p.S1600T |
| 240 | VUS | pm1 pm2 pp3 | *CKMT1A* | NM_001015001.2:exon7:c.796G>T:p.D266Y |
| 241 | VUS | pm2 pp3 | *TRPM7* | NM_017672.5:exon12:c.1376T>C:p.I459T |
| 242 | VUS | pm1 pm2 | *MYO1E* | NM_004998.3:exon24:c.2702A>C:p.Q901P |
| 243 | VUS | pm2 | *IREB2* | NM_004136.3:exon20:c.2477A>T:p.D826V |
| 244 | Benign | ba1 bp4 | *ADAMTS7* | NM_014272.4:exon19:c.3862G>A:p.G1288R |
| 245 | VUS | pm2 bp4 | *ADAMTSL3* | NM_207517.2:exon3:c.88C>T:p.P30S |
| 246 | VUS | pm1 pm2 | *TICRR* | NM_152259.3:exon12:c.2339C>G:p.S780C |
| 247 | VUS | pm1 pm2 bp4 | *LRRC28* | NM_144598.4:exon8:c.755G>A:p.R252Q |
| 248 | VUS | pm2 bp4 | *OR4F15* | NM_001001674.1:exon1:c.917C>T:p.A306V |
| 249 | VUS | pm2 pp3 | *C1QTNF8* | NM_207419.3:exon4:c.308G>A:p.G103E |
| 250 | Likely pathogenic | pm1 pm2 ps1 pp3 | *TSC2* | NM_000548.4:exon18:c.1939G>A:p.D647N |
| 251 | VUS | pm1 pm2 bp4 | *MEFV* | NM_000243.2:exon3:c.1129C>T:p.R377C |
| 252 | VUS | pm1 pm2 | *ERCC4* | NM_005236.2:exon11:c.2585A>G:p.N862S |
| 253 | VUS | pm2 | *SMG1* | NM_015092.4:exon61:c.10669C>G:p.Q3557E |
| 254 | VUS | bp4 | *ERI2* | NM_001142725.1:exon9:c.1349T>C:p.L450S |
| 255 | VUS | bp1 pm2 bp4 | *PALB2* | NM_024675.3:exon5:c.1955G>A:p.S652N |
| 256 | VUS | pp3 | *LAT* | NM_001014987.1:exon4:c.245C>T:p.P82L |
| 257 | VUS | pm1 pm2 bp4 | *CARMIL2* | NM_001013838.1:exon28:c.2858G>A:p.G953D |
| 258 | VUS | pm1 pm2 | *BCAR1* | NM_001170714.1:exon6:c.1825G>A:p.D609N |
| 259 | VUS | pm2 bp4 | *TLDC1* | NM_020947.3:exon2:c.19C>T:p.R7C |
| 260 | VUS | pm2 | *GSE1* | NM_001134473.2:exon7:c.1049C>T:p.P350L |
| 261 | VUS | bp4 | *PIEZO1* | NM_001142864.3:exon36:c.4813G>A:p.D1605N |
| 262 | VUS | pm2 | *HIC1* | NM_001098202.1:exon2:c.1048G>C:p.G350R |
| 263 | Benign | pm1 ba1 bp4 | *USP6* | NM_004505.3:exon6:c.373G>A:p.G125R |
| 264 | VUS | pm1 bp4 | *MFSD6L* | NM_152599.3:exon1:c.1195G>A:p.G399S |
| 265 | VUS | pm1 pm2 bp4 | *GLP2R* | NM_004246.2:exon4:c.499C>A:p.Q167K |
| 266 | VUS | pm1 pm2 | *MYH2* | NM_017534.5:exon3:c.151A>G:p.T51A |
| 267 | VUS | pm1 pm2 bp4 | *UTP6* | NM_018428.2:exon11:c.889G>A:p.A297T |
| 268 | VUS | pm2 | *ASIC2* | NM_183377.1:exon2:c.751G>A:p.E251K |
| 269 | VUS | pm1 | *UNC45B* | NM_173167.3:exon8:c.1109C>T:p.P370L |
| 270 | VUS | pm2 | *SLFN12* | NM_018042.4:exon2:c.547G>T:p.V183F |
| 271 | VUS | pm1 pm2 | *DDX52* | NM_007010.4:exon13:c.1591A>T:p.I531L |
| 272 | VUS | pm2 bp4 | *KRTAP4-3* | NM_033187.1:exon1:c.67A>T:p.S23C |
| 273 | VUS | pm1 pm2 pp3 | *STAT5A* | NM_003152.3:exon7:c.650G>A:p.R217H |
| 274 | VUS | pm1 bp4 | *SCPEP1* | NM_021626.2:exon8:c.721G>A:p.V241I |
| 275 | VUS | pm1 pm2 | *RPS6KB1* | NM_003161.3:exon15:c.1382C>T:p.A461V |
| 276 | VUS | pm1 pm2 pp3 | *SGSH* | NM_000199.3:exon4:c.397C>A:p.P133T |
| 277 | VUS | pm1 pm2 | *FSCN2* | NM_001077182.2:exon2:c.868G>A:p.E290K |
| 278 | VUS | pm2 bp4 | *ANKRD12* | NM_015208.4:exon9:c.5300C>T:p.P1767L |
| 279 | VUS | pm1 | *FHOD3* | NM_025135.4:exon10:c.1004C>G:p.P335R |
| 280 | VUS | pm2 bp6 | *TCF4* | NM_001083962.1:exon5:c.240G>A:p.M80I |
| 281 | VUS | pm2 | *PTBP1* | NM_002819.4:exon6:c.506C>T:p.A169V |
| 282 | VUS | bp4 | *PLPPR3* | NM_024888.2:exon7:c.2008T>C:p.S670P |
| 283 | VUS | pm1 pm2 | *GPX4* | UNKNOWN |
| 284 | VUS | pm2 | *APC2* | NM_005883.2:exon15:c.4376G>A:p.G1459D |
| 285 | VUS | pm1 pm2 | *THOP1* | NM_003249.4:exon6:c.748G>A:p.E250K |
| 286 | VUS | pm1 pm2 bp4 | *ZNF414* | NM_001146175.1:exon3:c.367G>A:p.V123I |
| 287 | Benign | pm1 ba1 bp4 | *MUC16* | NM_024690.2:exon56:c.40732A>G:p.I13578V |
| 288 | VUS | pm1 pm2 bp4 | *TYK2* | NM_003331.4:exon14:c.2003C>T:p.T668M |
| 289 | VUS | pm2 | *ZNF653* | NM_138783.3:exon4:c.713C>G:p.T238S |
| 290 | VUS | pm1 bp4 | *ZNF440* | NM_152357.2:exon4:c.1031T>C:p.I344T |
| 291 | VUS | pm1 pm2 pp3 | *CASP14* | NM_012114.2:exon4:c.350G>T:p.C117F |
| 292 | VUS | pm2 | *CPAMD8* | NM_015692.2:exon41:c.5458G>A:p.D1820N |
| 293 | VUS | pm1 pm2 | *SLC27A1* | NM_198580.2:exon6:c.910T>C:p.C304R |
| 294 | VUS | pm1 pm2 | *PGLS* | NM_012088.2:exon5:c.728C>T:p.A243V |
| 295 | Likely pathogenic | pm1 pm2 ps1 pp3 | *JAK3* | NM_000215.3:exon16:c.2062A>T:p.I688F |
| 296 | VUS | pm2 bp4 | *KIAA1683* | NM_001145304.1:exon3:c.2606A>G:p.Q869R |
| 297 | VUS | pm1 pm2 pp3 | *ARMC6* | NM_033415.3:exon5:c.841C>T:p.R281C |
| 298 | VUS | pm2 bp4 | *RFXANK* | NM_003721.3:exon3:c.82G>A:p.G28R |
| 299 | VUS | bp4 | *ZNF254* | NM_203282.3:exon1:c.10C>T:p.P4S |
| 300 | VUS | ps1 | *AXL* | NM_021913.4:exon11:c.1343G>T:p.W448L |
| 301 | VUS | pm2 | *CLPTM1* | NM_001294.3:exon10:c.1279G>A:p.V427I |
| 302 | VUS | pm2 | *SNRNP70* | NM_003089.5:exon10:c.1181G>A:p.G394D |
| 303 | VUS | pm1 | *PIH1D1* | NM_017916.2:exon8:c.727C>T:p.R243C |
| 304 | VUS | pm2 bp4 | *AKT1S1* | NM_001278160.1:exon4:c.568G>A:p.V190I |
| 305 | VUS | pm1 pm2 bp4 | *KLK7* | NM_001243126.1:exon4:c.499C>A:p.Q167K |
| 306 | VUS | pm1 pm2 pp3 | *KLK14* | NM_022046.5:exon7:c.761G>T:p.C254F |
| 307 | VUS | pm1 pm2 | *FPR3* | NM_002030.4:exon2:c.256G>A:p.A86T |
| 308 | VUS | pm2 | *LENG8* | NM_052925.3:exon8:c.1010G>A:p.R337Q |
| 309 | VUS | pm2 pp3 | *TNNI3* | NM_000363.4:exon6:c.298C>G:p.L100V |
| 310 | VUS | pm2 bp4 | *ZNF416* | NM_017879.1:exon4:c.370G>C:p.G124R |
| 311 | Likely pathogenic | pm1 pm2 ps1 pp3 | *TGM6* | NM_198994.2:exon10:c.1528G>C:p.D510H |
| 312 | VUS | pm1 pm2 | *MCM8* | NM_032485.5:exon8:c.832C>T:p.R278C |
| 313 | VUS | pm2 pp3 | *FOXS1* | NM_004118.3:exon1:c.511G>T:p.G171W |
| 314 | VUS | pm1 pm2 bp4 | *DNMT3B* | NM_006892.3:exon15:c.1610G>A:p.R537Q |
| 315 | VUS | pm2 | *RALY* | NM_016732.2:exon4:c.322A>G:p.I108V |
| 316 | VUS | pm1 pm2 bp4 | *FAM65C* | NM_080829.3:exon2:c.67G>T:p.A23S |
| 317 | VUS | pm1 pm2 | *COL20A1* | NM_020882.2:exon14:c.1678C>G:p.P560A |
| 318 | VUS | pm2 | *TFF2* | NM_005423.4:exon4:c.379T>C:p.C127R |
| 319 | VUS | pm2 bp4 | *TRPM2* | NM_003307.3:exon8:c.1152C>A:p.S384R |
| 320 | VUS | pm2 pp3 | *TRPM2* | NM_003307.3:exon13:c.2057C>G:p.A686G |
| 321 | VUS | pm1 pm2 pp3 | *TUBA8* | NM_018943.2:exon3:c.265C>A:p.P89T |
| 322 | VUS | pm1 pm2 | *CLTCL1* | NM_007098.3:exon6:c.938C>G:p.S313C |
| 323 | VUS | pm1 pm2 bp4 | *AIFM3* | NM_144704.2:exon3:c.151A>C:p.T51P |
| 324 | VUS | pm2 bp4 | *SLC7A4* | NM_004173.2:exon3:c.1417A>G:p.S473G |
| 325 | VUS | pm2 | *TMEM191C* | NM_001207052.1:exon3:c.442C>G:p.Q148E |
| 326 | VUS | bp4 | *LRRC75B* | NM_207644.2:exon2:c.242C>T:p.P81L |
| 327 | VUS | pm2 | *BCRP3* | . |
| 328 | VUS | pm2 pm3_supporting | *NEFH* | . |
| 329 | VUS | pm2 bp4 | *PRR14L* | NM_173566.2:exon4:c.4459T>C:p.C1487R |
| 330 | VUS | pm2 bp4 | *TRIOBP* | NM_001039141.2:exon7:c.2149C>A:p.Q717K |
| 331 | VUS | pm2 bp6 | *DMD* | NM_004006.2:exon28:c.3794G>C:p.W1265S |
| 332 | VUS | pm1 pm2 | *PSMD10* | NM_002814.3:exon5:c.655A>T:p.I219L |
| 333 | VUS | pm2 pp3 | *COL4A5* | NM_000495.4:exon3:c.220C>T:p.R74W |
| 334 | VUS | pm2 bp4 | *MAGEA4* | NM_001011548.1:exon3:c.940G>A:p.E314K |
| 335 | VUS | pvs1_moderate pm2 pp3 | *SLC6A6* | NM_001134367.3:NA:c.230-1del:.,NM_001134367.3:NA:c.230-1del:. |
| 336 | VUS | pm2 | *FAM166B* | NM_001164310.2:exon5:c.714_715insT:p.P239Sfs*2 |
| 337 | VUS | pm2 | *UBQLN3* | NM_017481.3:exon2:c.179_192del:p.Q60Lfs*10 |
| 338 | VUS | pm2 pm3_supporting | *LOC283710* | NM_001243538.2:exon2:c.76del:p.R26Gfs*101 |
| 339 | Likely pathogenic | pvs1_moderate pm2 pm1 pp3 | *ACAN* | NM_013227.3:exon16:c.7230del:p.F2410Lfs*9 |
| 340 | VUS | pm2 | *ERN2* | NM_033266.3:exon17:c.2093_2096del:p.D698Vfs*64 |
| 341 | VUS | pm2 | *ABCC3* | NM_003786.3:exon17:c.2226dup:p.E743Rfs*8 |
| 342 | VUS | pm2 | *ZNF528* | NM_032423.2:exon7:c.1225dup:p.Y409Lfs*11 |
| 343 | VUS | pm2 | *PRIM2* | UNKNOWN |
| 344 | VUS | pm2 | *MROH5* | UNKNOWN |
